# The relationship between tumor budding and survival of patients with breast cancer: A meta-analysis

**DOI:** 10.17305/bb.2024.11103

**Published:** 2024-12-01

**Authors:** Hongjie Xu, Dajun Wei

**Affiliations:** 1 Department of Oncology, Affiliated Hospital of Beihua University, Jilin City, Jilin Province, China; 2 Department of Cardiology, Affiliated Hospital of Beihua University, Jilin City, Jilin Province, China

**Keywords:** Tumor budding, breast cancer, BC, survival, prognosis, meta-analysis

## Abstract

Tumor budding has been proposed as a potential prognostic marker in various cancers, but its association with survival outcomes in breast cancer (BC) remains unclear. This meta-analysis aimed to clarify the relationship between tumor budding and survival outcomes in patients with BC. A comprehensive literature search was conducted in PubMed, EMBASE, and Web of Science. Cohort studies examining the association between tumor budding and overall survival (OS) and progression-free survival (PFS) in BC patients were included. Hazard ratios (HRs) and 95% confidence intervals (CIs) were pooled using a random-effects model to account for potential heterogeneity. Eleven cohort studies, including 2828 patients, met the inclusion criteria. High tumor budding was significantly associated with poorer OS (HR ═ 1.89, 95% CI ═ 1.37–2.60, *P* < 0.001) and PFS (HR ═ 1.89, 95% CI ═ 1.32–2.71, *P* < 0.001). Subgroup analyses revealed a stronger association in studies where high tumor budding was defined as ≥ 10 buds/high-power field (HPF) compared to those with lower cutoffs. Sensitivity analyses confirmed the robustness of the findings. This meta-analysis demonstrates that high tumor budding is associated with significantly worse OS and PFS in BC patients, underscoring its prognostic significance. These findings suggest tumor budding could be a valuable marker in clinical assessments, and further research is needed to standardize its evaluation criteria in BC.

## Introduction

Breast cancer (BC) is the most commonly diagnosed malignancy and a leading cause of cancer-related mortality among women worldwide [1, 2]. Despite advancements in early detection and treatment, BC remains a significant public health burden due to its high prevalence and variability in patient outcomes [3]. Survival rates for BC patients can vary widely based on factors, such as tumor characteristics, treatment modalities, and patient demographics [4–6]. Therefore, identifying reliable prognostic markers and risk factors associated with poor survival is crucial for improving patient management and outcomes. Tumor budding, defined as the presence of isolated single cells or small clusters of up to four cells at the invasive front of tumors [7–9], has emerged as a potential prognostic marker in various cancers, including colorectal [10], pancreatic [11], and esophageal cancers [12]. The mechanisms underlying tumor budding involve epithelial–mesenchymal transition (EMT), where epithelial cells acquire mesenchymal traits, enhancing their migratory and invasive capabilities [13]. This process contributes to tumor progression, metastasis, and resistance to therapy, ultimately leading to a poorer prognosis [14, 15]. In BC, tumor budding is believed to facilitate metastatic spread by enabling cancer cells to dissociate from the primary tumor mass and invade surrounding tissues and distant organs [16]. Evidence has linked tumor budding to several malignant characteristics of BC, such as higher tumor grade, increased lymphovascular invasion, and reduced hormone receptor expression [17, 18].

However, previous studies evaluating the association between tumor budding and BC patient survival have shown inconsistent results [19]. Given the potential of tumor budding as a prognostic marker, this meta-analysis aims to systematically evaluate and quantify the relationship between tumor budding and survival outcomes in BC patients. By synthesizing data from multiple cohort studies, we aim to provide a comprehensive understanding of how tumor budding impacts overall survival (OS) and progression-free survival (PFS) in BC patients. This analysis could inform clinical decision making and guide future research on targeted interventions for patients at higher risk of poor outcomes due to tumor budding.

## Materials and methods

This meta-analysis adhered to the Preferred Reporting Items for Systematic Reviews and Meta-Analyses (PRISMA 2020) guidelines [20, 21] and the Cochrane Handbook for Systematic Reviews and Meta-Analyses [22] throughout its design, data collection, statistical analysis, and interpretation of the results.

### Data sources and search strategy

A comprehensive literature search was performed in PubMed, EMBASE, and Web of Science to identify relevant cohort studies published from database inception to June 22, 2024. The search strategy included the combined terms of (1) “budding” OR “sprouting” OR “bud” OR “buds” OR “tumor cell dissociation”; (2) “breast cancer”; and (3) “mortality” OR “survival” OR “recurrence” OR “death” OR “prognosis” OR “progression” OR “metastasis.” The detailed search strategy for each database is shown in Supplemental File 1. Only studies published in English as full-length articles in peer-reviewed journals were included. Additionally, the reference lists of the identified articles and relevant reviews were screened to ensure comprehensive coverage.

### Study selection

Studies were included if they met the following criteria designed according to the PICOS model:

- **P** (patients): Patients with a confirmed diagnosis of BC, without limitations on cancer stage or treatment.
- **I** (exposure): Patients with high tumor budding at enrollment. The methods for evaluating tumor budding and the cutoff values for defining high tumor budding were consistent with those used in the included studies.
- **C** (comparison): Patients with low tumor budding at enrollment.
- **O** (outcome): Reported at least one of the following outcomes compared between patients with high vs low tumor budding at baseline: OS or PFS. OS was defined as the time from enrollment to death from any cause. PFS was defined as the interval from enrollment to the first BC recurrence or progression.
- **S** (study design): Longitudinal studies, including cohort studies, nested case-control studies, and post hoc analyses of clinical trials.

The exclusion criteria included reviews, editorials, meta-analyses, preclinical studies, cross-sectional studies, studies involving patients with cancers other than BC, and studies that did not report survival outcomes. For studies with overlapping patient populations, the study with the largest sample size was chosen for the meta-analysis.

### Quality evaluation and data extraction

Two authors independently performed the literature search, study identification, quality evaluation, and data collection. Disagreements were resolved by consensus between the two authors. Study quality was assessed using the Newcastle–Ottawa Scale (NOS) [23], which evaluates studies based on the selection of the study population, comparability between groups, and measurement of exposure. NOS scores ranged from 0 to 9, with higher scores indicating better study quality. A score of 7–9 was considered high quality [23]. Data extracted from each study included study details (authors, year, design, and country), patient characteristics (diagnosis, sample size, age, tumor stage, and main treatments), methods for evaluating tumor budding and cutoffs for defining high tumor budding, follow-up duration, outcomes reported, and variables adjusted for in evaluating the association between tumor budding and survival outcomes of BC patients.

### Statistical analysis

The association between tumor budding and survival outcomes in BC was summarized using hazard ratios (HRs) and 95% confidence intervals (CIs). HRs and standard errors (SEs) were calculated from 95% CIs or *P* values, and logarithmic transformation was applied to stabilize and normalize variance. Study heterogeneity was assessed using the Cochrane *Q* test and *I*^2^ statistics, with *I*^2^ > 50% indicating significant statistical heterogeneity [24]. Given the clinical variability among the studies (e.g., patient characteristics, treatments, cutoffs for defining high tumor budding, and follow-up durations), a random-effects model using the inverse-variance approach with DerSimonian and Laird was used for all meta-analyses to account for between-study heterogeneity [22]. Sensitivity analyses were performed by sequentially omitting each study to test the robustness of the results. A predefined subgroup analysis was conducted to evaluate how study characteristics, such as country, cutoff for defining high tumor budding, mean age, follow-up duration, and analytic models (multivariate or univariate analysis), affected the results. Publication bias was initially assessed using funnel plots and visual inspection of symmetry [25], followed by Egger’s regression test [25]. Statistical analyses were performed using RevMan (Version 5.1; Cochrane Collaboration, Oxford, UK) and Stata (version 12.0; Stata Corporation, College Station, TX, USA), with a two-sided *P* value < 0.05 considered statistically significant.

## Results

### Database search and study inclusion

**Figure 1.**
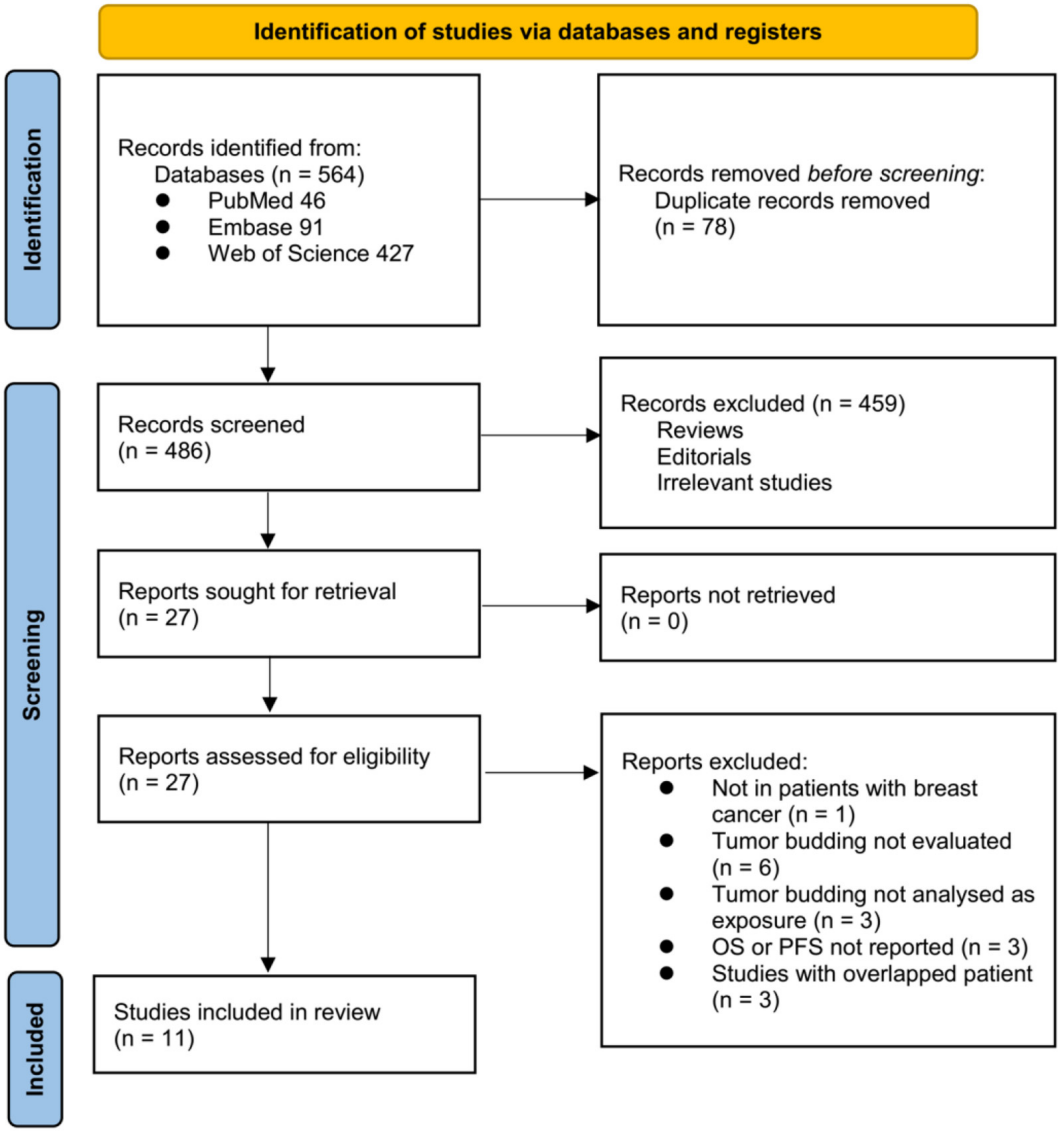
**The flowchart shows database search process and study inclusion.** PFS: Progression-free survival; OS: Overall survival.

The study inclusion process is illustrated in Figure 1. Initially, 564 potentially relevant records were retrieved from the three databases, of which 78 were removed due to duplication. After screening titles and abstracts, 459 studies were further excluded, primarily because they were not relevant to the meta-analysis. Two independent authors reviewed the full texts of the remaining 27 records and excluded 16 additional studies for reasons detailed in Figure 1. Ultimately, 11 cohort studies were deemed suitable for quantitative analysis [26–36].

### Characteristics of the included studies

Table 1 summarizes the characteristics of the included studies. The meta-analysis included 11 retrospective cohort studies [26–36], conducted in China, the United States, Turkey, Canada, Japan, Portugal, and Iran. One study reported data on different histological types of BC (ER+/HER2- and triple-negative BC [TNBC]), and these datasets were included independently in the meta-analysis [27]. Overall, 2828 patients with BC were included, with mean ages ranging from 52 to 63 years. Eight studies included patients with stage I-III BC [26–31, 33, 34], while two studies included patients with stage I-IV BC [35, 36]. Surgical resection was the main treatment in ten studies [26–35]. Tumor budding analysis was performed using Hematoxylin and Eosin (H&E) staining in ten studies [26–30, 32–36], while one study used pan-cytokeratin immunohistochemistry [31]. The microscopic magnifications were either 200× [26–34, 36] or 400× [35]. The cutoff for defining high tumor budding was 5 buds per high-power field (HPF) in seven studies [26–28, 32, 33, 35, 36], 7 buds/HPF in one study [29], 8 buds/HPF in another [31], and 10 buds/HPF in two studies [30, 34]. The median follow-up durations ranged from 7.2–101 months. OS was reported in nine studies [26–30, 32, 34–36], and PFS was reported in eight studies [26, 27, 29–31, 33–35]. Multivariate analyses adjusting for variables such as age, tumor grade, stage, and lymphovascular invasion were performed in six studies [26, 27, 29–31, 34], while univariate analyses were performed in the remaining five studies [28, 32, 33, 35, 36]. The NOS scores of the included studies ranged from six to nine stars, indicating moderate-to-high study quality (Table 2).

**Table 1:** Summary of the characteristics of the included studies

**Study**	**Location**	**Design**	**Diagnosis**	**Sample size**	**Mean age (years)**	**Stage**	**Main treatment**	**Stains**	**Microscopic magnification**	**Cutoff values**	**Median follow-up duration (months)**	**Outcomes**	**Variables adjusted**
Sun, 2014	China	RC	Operable invasive ductal BC	146	52	I–III	Surgical resection	H&E	×200	≥5 buds/HPF	46	OS and PFS	Age, tumor stage, grade, LVI, ER/PR expression, and HER-2 expression
Li, 2017 ER+/HER2-	The US	RC	ER+/HER2- BC	244	55.2	I–III	Surgical resection	H&E	×200	≥5 buds/HPF	72.7	OS and PFS	Age, tumor size, stage, and LVI
Li, 2017 TNBC	The US	RC	TNBC	131	56.2	I–III	Surgical resection	H&E	×200	≥5 buds/HPF	7.2	OS and PFS	Age, tumor size, stage, and LVI
Okcu, 2021	Turkey	RC	Operable invasive ductal BC	311	57.2	I–III	Surgical resection	H&E	×200	≥7 buds/HPF (ROC curve analysis derived)	47	OS and PFS	Age, tumor stage, grade, LVI, ER/PR expression, HER-2 expression, and Ki-67 group
Mozarowski, 2021	Canada	RC	Operable BC	75	58.9	I–III	Neo-adjuvant therapy and surgical resection	H&E	×200	≥5 buds/HPF	50	OS	None
Xiang, 2022	China	RC	Invasive BC	229	NR	I–III	Surgical resection	Pan-cytokeratin IHC	×200	≥8 buds/HPF (X-Tile derived)	43.5	PFS	Age, tumor stage, grade, and HER2 expression
Hiratsuka, 2022	Japan	RC	Operable invasive ductal BC	855	56	I–III	Surgical resection	H&E	×200	≥10 buds/HPF	58	OS and PFS	Age, tumor stage, grade, LVI, ER/PR expression, HER-2 expression, Ki-67 group, and anticancer treatment
Silva, 2023	Portugal	RC	Early BC	100	63	I–III	Lumpectomy or mastectomy	H&E	×200	≥5 buds/HPF	101	PFS	None
Ozer, 2023	Turkey	RC	Invasive BC	198	56.2	NR	Surgical resection	H&E	×200	≥5 buds/HPF	39.6	OS	None
Hou, 2024	China	RC	TNBC	118	NR	I–III	Surgical resection	H&E	×200	≥10 buds/HPF	40	OS and PFS	Age, tumor size, grade, stage, and Ki-67 groups
Ranaee, 2024	Iran	RC	Invasive BC	150	54	I–IV	NR	H&E	×200	≥5 buds/HPF	30	OS	None
Ozsen, 2024	Turkey	RC	Invasive BC	271	54.8	I–IV	Surgical resection	H&E	×400	≥5 buds/HPF	60	OS and PFS	None

BC: Breast cancer; ER: Estrogen receptor; HER-2: Human epidermal growth factor receptor 2; H&E: Hematoxylin and Eosin; HPF: High-power field; IHC: Immunohistochemistry; LVI: Lymphovascular invasion; OS: Overall survival; PFS: Progression-free survival; PR: Progesterone receptor; RC: Retrospective cohort; ROC: Receiver operating characteristic; TNBC: Triple-negative breast cancer; NR: Not reported.

**Table 2:** Study quality evaluation via NOS

**Study**	**Representa -tiveness of the exposed cohort**	**Selection of the non-exposed cohort**	**Ascertainment of exposure**	**Outcome not present at baseline**	**Control for age**	**Control for other confounding factors**	**Assessment of outcome**	**Enough long follow-up duration**	**Adequacy of follow-up of cohorts**	**Total**
Sun, 2014	0	1	1	1	1	1	1	1	1	8
Li, 2017 ER+/HER2--	0	1	1	1	1	1	1	1	1	8
Li, 2017 TNBC	0	1	1	1	1	1	1	0	1	7
Okcu, 2021	0	1	1	1	1	1	1	1	1	8
Mozarowski, 2021	0	1	1	1	0	0	1	1	1	6
Xiang, 2022	0	1	1	1	1	1	1	1	1	8
Hiratsuka, 2022	1	1	1	1	1	1	1	1	1	9
Silva, 2023	0	1	1	1	0	0	1	1	1	6
Ozer, 2023	0	1	1	1	0	0	1	1	1	6
Hou, 2024	0	1	1	1	1	1	1	1	1	8
Ranaee, 2024	0	1	1	1	0	0	1	1	1	6
Ozsen, 2024	0	1	1	1	0	0	1	1	1	6

NOS: Newcastle–Ottawa scale.

### Association between tumor budding and OS of patients with BC

**Figure 2.**
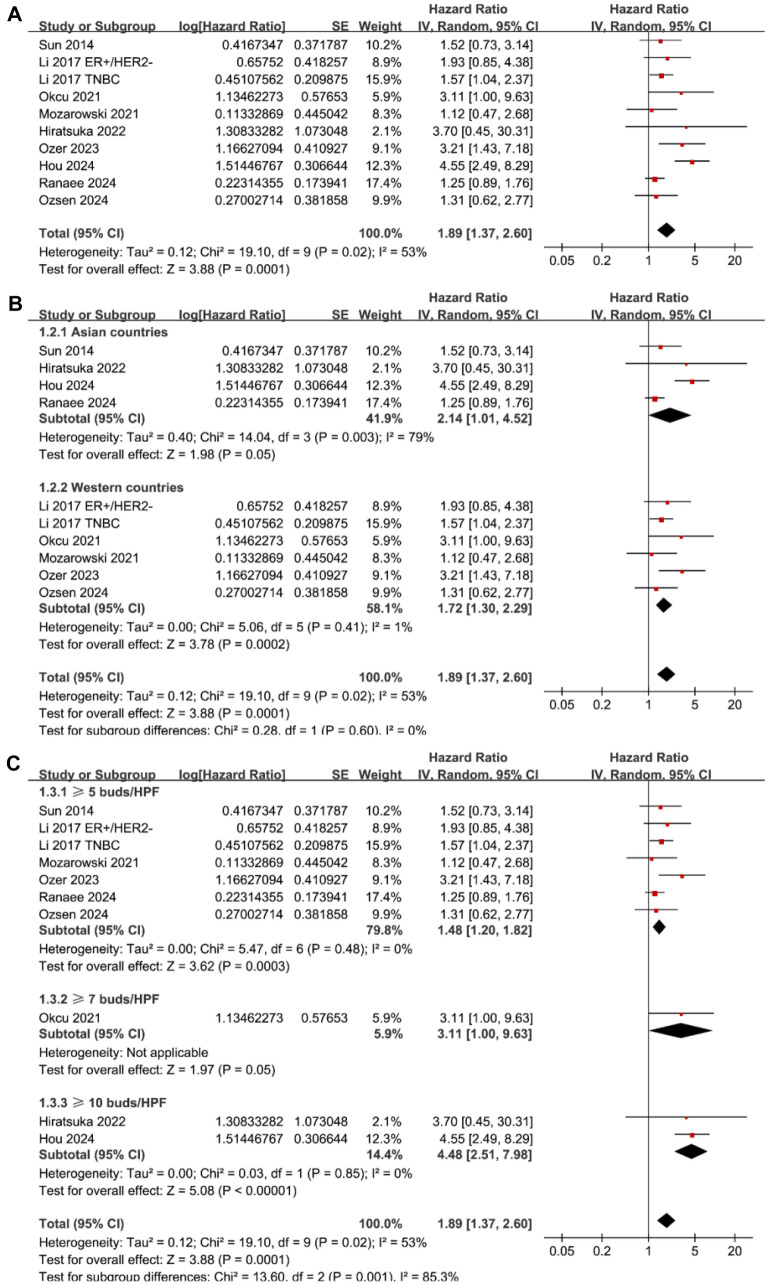
**Forest plots for the meta-analysis of the association between tumor budding and OS in patients with BC.** (A) Forest plots for the overall meta-analysis; (B) Forest plots for the subgroup analysis according to the study country; (C) Forest plots for the subgroup analysis according to the cutoffs for defining a high tumor budding. BC: Breast cancer; OS: Overall survival.

**Figure 3.**
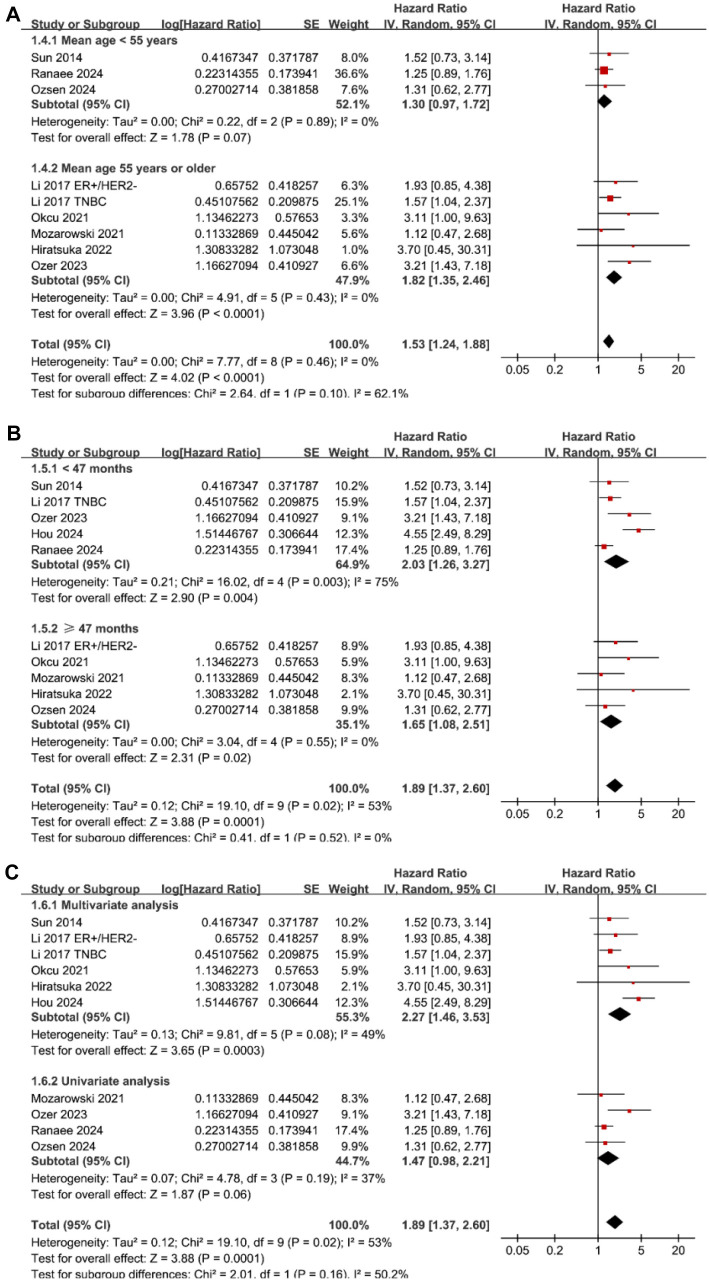
**Forest plots for subgroup analyses of the association between tumor budding and OS of patients with BC.** (A) Forest plots for subgroup analysis according to mean age of the patients; (B) Forest plots for subgroup analysis according to follow-up duration; (C) Forest plots for subgroup analysis according to analytic models. BC: Breast cancer; OS: Overall survival.

Pooled results from ten datasets across nine studies [26–30, 32, 34–36] revealed that high tumor budding at enrollment was associated with poor OS in patients with BC compared to low tumor budding (HR ═ 1.89, 95% CI ═ 1.37–2.60, *P* < 0.001; Figure 2A) with moderate heterogeneity (*I*^2^ ═ 53%). Sensitivity analysis, where one study was omitted at a time, did not significantly alter the results (HR: 1.53–2.05, all *P* < 0.05). Subgroup analyses showed similar results in studies from both Asian and Western countries (*P* for subgroup difference ═ 0.60; Figure 2B). Interestingly, subgroup analysis suggested a stronger association in studies with a high tumor budding cutoff of ≥ 10 buds/HPF (HR ═ 4.48, 95% CI ═ 2.51–7.98), compared to studies using cutoffs of ≥ 7 buds/HPF (HR ═ 3.11, 95% CI ═ 1.00–9.63) and ≥ 5 buds/HPF (HR ═ 1.48, 95% CI ═ 1.20–1.82), which fully explained the heterogeneity (*P* for subgroup difference ═ 0.001; Figure 2C). Further subgroup analyses based on mean age (*P* for subgroup difference ═ 0.10; Figure 3A), follow-up duration (*P* for subgroup difference ═ 0.52; Figure 3B), and analytic models (*P* for subgroup difference ═ 0.16; Figure 3C) yielded similar results.

### Association between tumor budding and PFS of patients with BC

**Figure 4.**
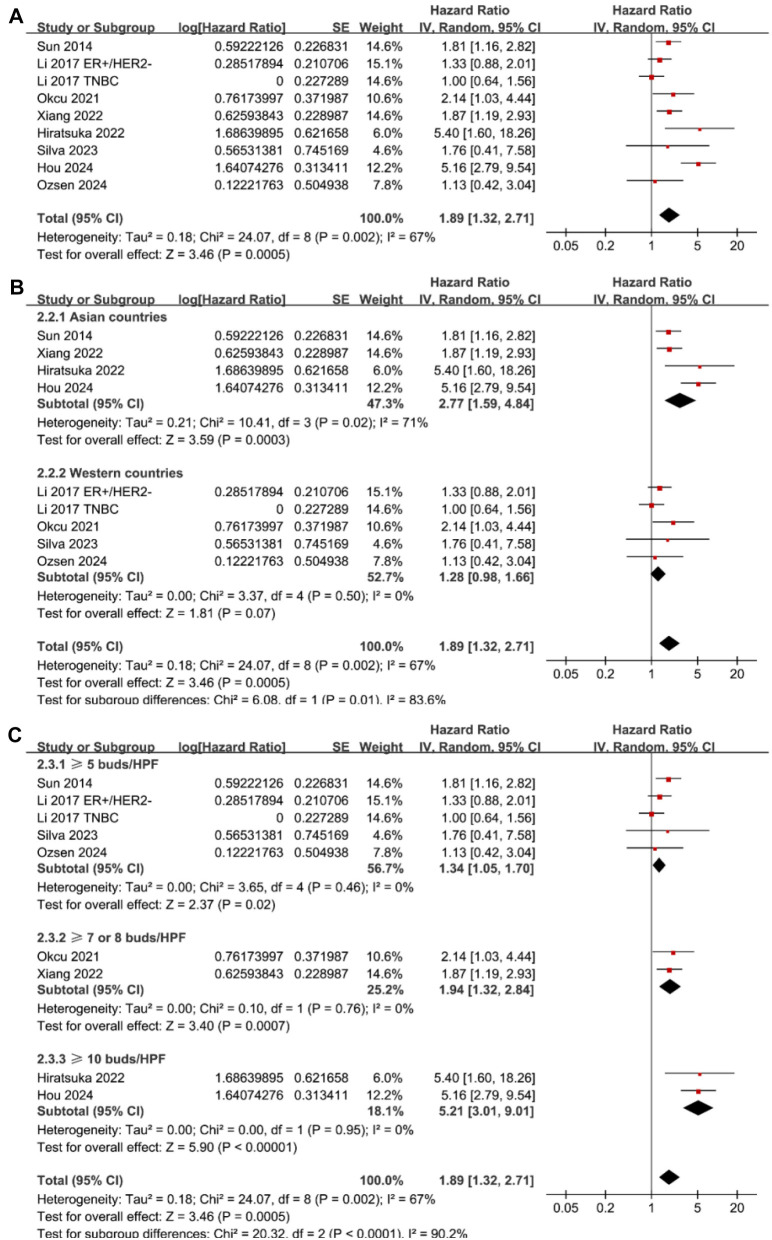
**Forest plots for the meta-analysis of the association between tumor budding and PFS in patients with BC.** (A) Forest plots for the overall meta-analysis; (B) Forest plots for the subgroup analysis according to the study country; (C) Forest plots for the subgroup analysis according to the cutoffs for defining a high tumor budding. BC: Breast cancer; PFS: Progression-free survival.

The meta-analysis of nine datasets from eight studies [26, 27, 29–31, 33–35] indicated that patients with high tumor budding had significantly poorer PFS compared to those with low tumor budding (HR ═ 1.89, 95% CI ═ 1.32–2.71, *P* < 0.001; Figure 4A). Sensitivity analysis, excluding one dataset at a time, produced similar results (HR: 1.58–2.10, *P* < 0.05).

**Figure 5.**
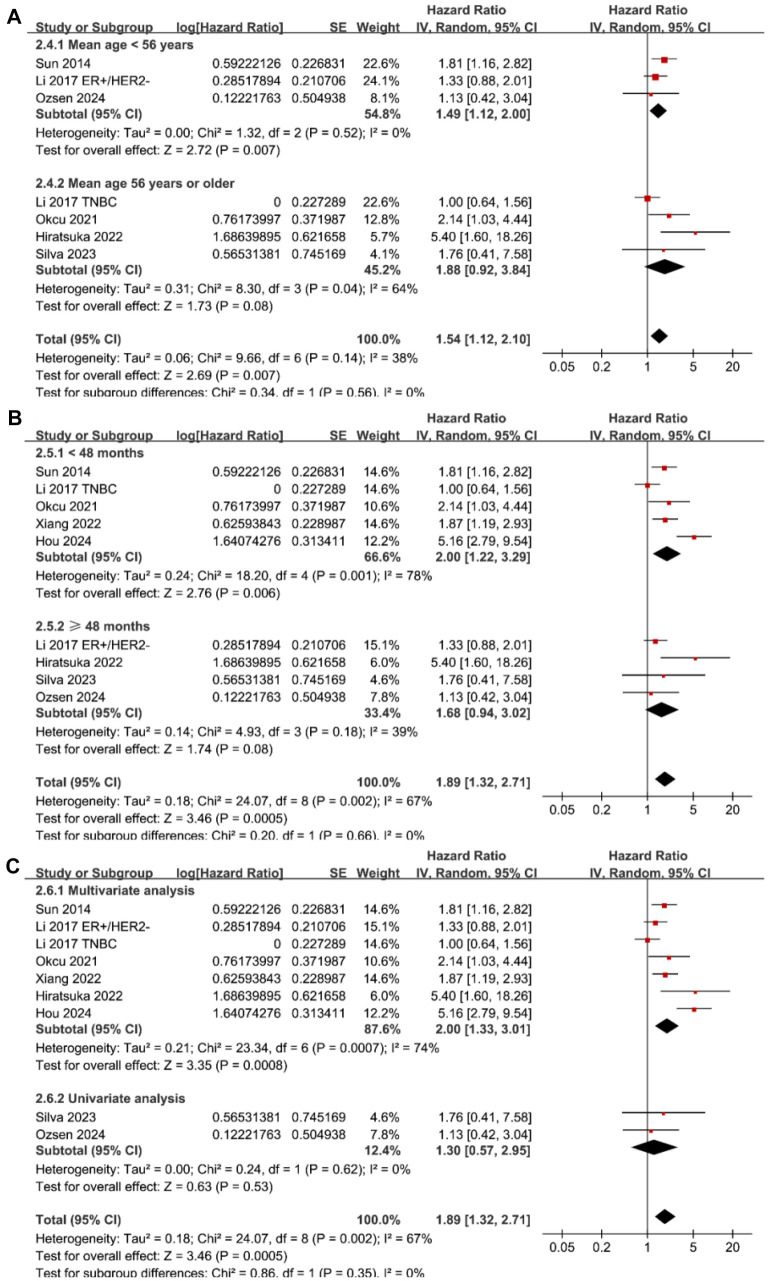
**Forest plots for subgroup analyses of the association between tumor budding and PFS of patients with BC.** (A) Forest plots for subgroup analysis according to mean age of the patients; (B) Forest plots for subgroup analysis according to follow-up duration; (C) Forest plots for subgroup analysis according to analytic models. BC: Breast cancer; PFS: Progression-free survival.

Subgroup analysis revealed a stronger association between high tumor budding and poor PFS in studies conducted in Asian countries compared to non-Asian countries (HR: 2.77 vs 1.28, *P* for subgroup difference ═ 0.01; Figure 4B), though significant heterogeneity was noted among Asian studies (*I*^2^ ═ 71%). Additionally, subgroup analysis based on tumor budding cutoffs demonstrated a stronger association in studies using a cutoff of ≥ 10 buds/HPF (HR ═ 5.21, 95% CI ═ 3.01–9.01) compared to cutoffs of ≥ 7 or 8 buds/HPF (HR ═ 1.94, 95% CI ═ 1.32–2.84) and ≥ 5 buds/HPF (HR ═ 1.34, 95% CI ═ 1.05–1.70), fully explaining the heterogeneity (*P* for subgroup difference < 0.001; Figure 4C). Further subgroup analyses based on mean age (*P* for subgroup difference ═ 0.56; Figure 5A), follow-up duration (*P* for subgroup difference ═ 0.66; Figure 5B), and analytic models (*P* for subgroup difference ═ 0.35; Figure 5C) showed consistent results.

### Publication bias

**Figure 6.**
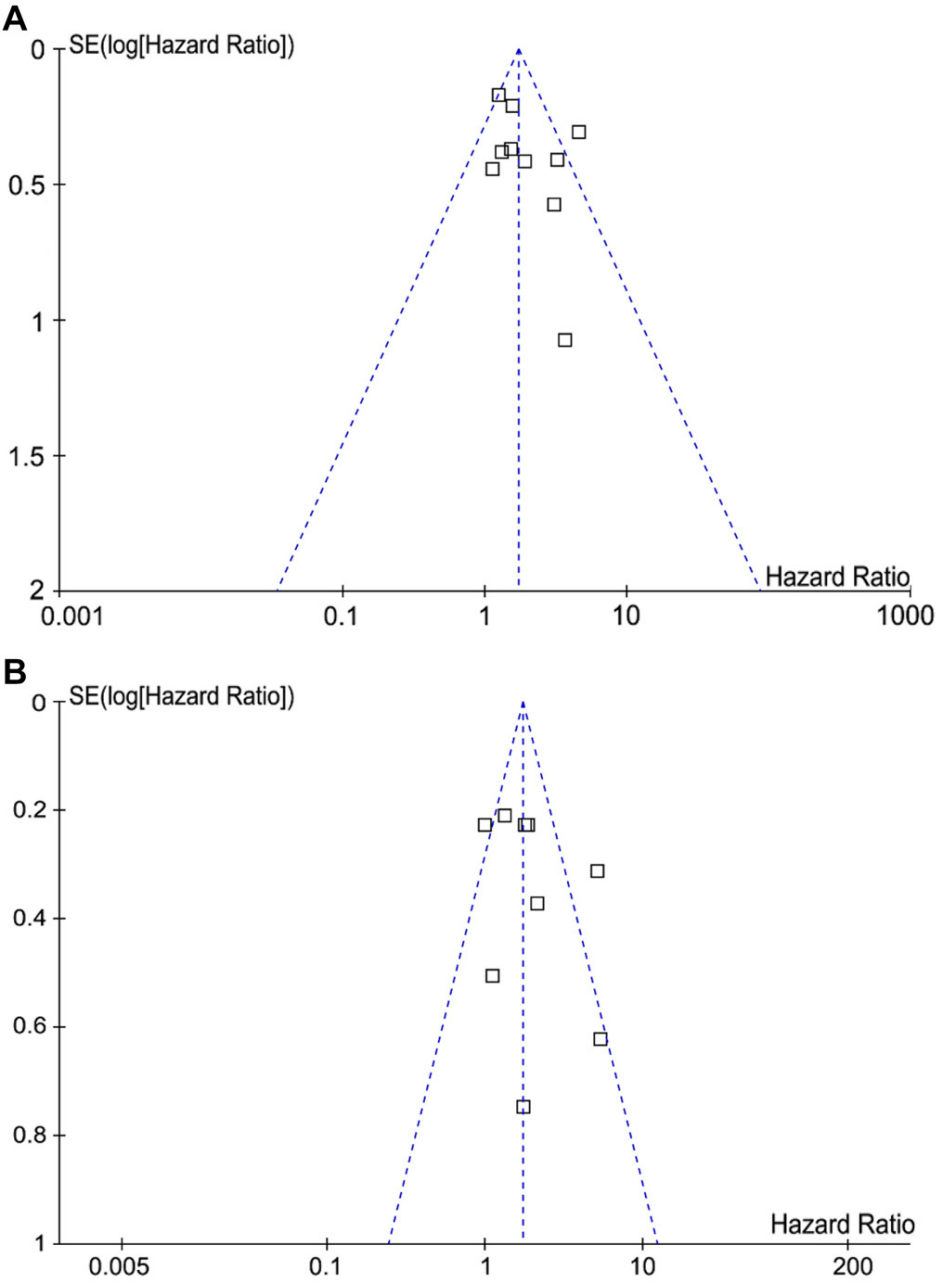
**Funnel plots for the meta-analysis of the associations of tumor budding with OS and PFS in patients with BC.** (A) Funnel plots for the outcome of OS; (B) Funnel plots for the outcome of PFS. BC: Breast cancer; OS: Overall survival; PFS: Progression-free survival.

Funnel plots for the associations between tumor budding and OS and PFS in BC patients appeared symmetrical, suggesting minimal publication bias (Figure 6A and 6B). Egger’s tests further confirmed low publication bias for OS and PFS (*P* ═ 0.52 and 0.66, respectively).

## Discussion

This meta-analysis aimed to clarify the relationship between tumor budding and survival outcomes in BC patients. The findings revealed a significant association between high tumor budding and poorer OS and PFS. Specifically, patients with high tumor budding had an approximately 89% increased risk of mortality and a similar increase in the risk of disease progression compared to those with low tumor budding. These results highlight the prognostic value of tumor budding in BC and suggest that it could serve as an important marker for identifying patients at higher risk of adverse outcomes. Several potential mechanisms might explain the link between high tumor budding and poor survival in BC. Tumor budding is closely related to epithelial–mesenchymal transition (EMT), a process in which epithelial cells lose their cell–cell adhesion properties and gain migratory and invasive capabilities [37]. EMT is driven by several molecular pathways, including the activation of transcription factors, such as Snail, Slug, and Twist, which repress E-cadherin expression and promote the expression of mesenchymal markers like N-cadherin and vimentin [38]. Additionally, signaling pathways involving TGF-β, Wnt/β-catenin, and Notch are known to play crucial roles in EMT and tumor budding [38]. These pathways facilitate the detachment of tumor cells from the primary mass, enhancing their invasive potential and contributing to metastasis and therapy resistance [39, 40]. Consequently, the presence of tumor budding reflects a more aggressive tumor phenotype, which could lead to poorer survival outcomes in BC patients. The subgroup analyses provided further insights into the impact of tumor budding on survival outcomes. Interestingly, the cutoff used to define high tumor budding significantly influenced the strength of the association with OS and PFS, which fully explained the source of statistical heterogeneity. Studies that used a cutoff of ≥ 10 buds/HPF reported a much stronger association between high tumor budding and poor survival compared to studies with lower cutoffs. This variation might be due to differences in the sensitivity and specificity of detecting truly aggressive tumor cells. A higher cutoff could better capture the most aggressive and clinically relevant tumor budding, leading to a more pronounced impact on survival outcomes. Conversely, lower cutoffs might include less aggressive tumor cells, diluting the association with poor prognosis. Similarly, a study in colorectal cancer patients demonstrated that tumor budding with ≥ 10 tumor buds/HPF was associated with a more than twofold increased risk of cancer-specific death, whereas the association was not significant for patients with 1–9 tumor buds/HPF [41]. These findings underscore the importance of standardizing criteria for defining and assessing tumor budding to ensure consistency and comparability across studies. It is also important to consider the influence of study quality (as measured by NOS) on the outcomes of this meta-analysis. According to the NOS criteria, a score of 7–9 indicates good study quality [42]. The NOS scores of the included studies ranged from 6 to 9. Notably, studies with a score of 6 (indicating moderate quality) primarily used univariate analysis, while studies with scores of 7–9 (indicating high quality) utilized multivariate analysis. Our subgroup analysis revealed similar outcomes between univariate and multivariate analyses, further demonstrating consistent results across studies of both moderate and high quality.

### Limitations

The current meta-analysis has several strengths. First, it adhered to rigorous methodological standards, including a comprehensive literature search, well-defined inclusion criteria, and robust statistical analyses. The inclusion of studies from diverse geographic regions enhances the generalizability of the findings. Second, the meta-analysis only included cohort studies, which offer a longitudinal relationship between tumor budding and poor survival outcomes in BC patients [43]. Additionally, we performed multiple sensitivity and subgroup analyses to confirm the robustness of the findings and explore sources of heterogeneity. Specifically, positive results in subgroup analyses limited to multivariate studies suggest that the association between tumor budding and poor survival may be independent of potential confounding factors, such as age, tumor grade, or tumor stage, providing more reliable estimates of this relationship.

However, several limitations should be acknowledged. All included studies were retrospective cohort studies, which are subject to inherent biases such as selection bias and recall bias [44]. The heterogeneity in the methodologies used to assess tumor budding—including differences in staining techniques and cutoffs for defining high tumor budding—might have contributed to variability in the results. Despite using a random-effects model to account for between-study heterogeneity [45], the presence of significant heterogeneity in some subgroup analyses indicates that other unmeasured factors might influence the association between tumor budding and survival outcomes. Furthermore, as this is a meta-analysis of observational studies, the causation between high tumor budding and poor survival in BC cannot be definitively established based on the current results. Lastly, the potential influence of hormone receptor status [46] and BC subtypes [47] on the meta-analysis outcome could not be determined because stratified data by these factors were not commonly reported in the included studies. Further investigation is warranted in future studies.

### Clinical implications

From a clinical perspective, the findings of this meta-analysis emphasize the potential of tumor budding as a prognostic marker in BC. Identifying patients with high tumor budding could help stratify risk and guide treatment decisions. For instance, patients with high tumor budding might benefit from more aggressive therapeutic strategies and closer surveillance to improve their outcomes. Additionally, the results highlight the need for further research to validate the prognostic value of tumor budding in larger, prospective studies and to standardize tumor budding assessments in clinical practice. Future research should also explore the molecular mechanisms underlying the association between tumor budding and poor survival in BC. Investigating the role of EMT and related pathways in tumor budding could provide valuable insights into the biology of tumor progression and metastasis [48]. Moreover, it is crucial to assess whether integrating tumor budding assessment with other established prognostic markers, such as hormone receptor status and HER2 expression, could enhance the accuracy of risk stratification and personalized treatment approaches [49].

## Conclusion

In conclusion, this meta-analysis demonstrates that high tumor budding is significantly associated with poorer OS and PFS in patients with BC. The impact of the cutoff for defining high tumor budding on survival outcomes underscores the need for standardized assessment criteria. Future research should aim to validate these findings in larger, prospective studies and further elucidate the underlying molecular mechanisms to improve the management and outcomes of BC patients.

## Supplemental data

**Supplemental File 1. Detailed search strategy**

**PubMed (*n* ═ 46)**

((“budding”[All Fields] OR “sprouting”[All Fields] OR “bud”[All Fields] OR “buds”[All Fields] OR “tumor cell dissociation”[All Fields]) AND (“breast neoplasms”[MeSH Terms] OR “breast cancer”[All Fields]) AND (“mortality”[MeSH Terms] OR “survival”[MeSH Terms] OR “recurrence”[MeSH Terms] OR “death”[MeSH Terms] OR “prognosis”[MeSH Terms] OR “progression”[MeSH Terms] OR “metastasis”[MeSH Terms]))

**Embase (*n* ═ 91)**

(’budding’ OR ’sprouting’ OR ’bud’ OR ’buds’ OR ’tumor cell dissociation’) AND (’breast cancer’/exp OR ’breast cancer’) AND (’mortality’/exp OR ’survival’/exp OR ’recurrence’/exp OR ’death’/exp OR ’prognosis’/exp OR ’progression’/exp OR ’metastasis’/exp) AND [humans]/lim AND [clinical study]/lim AND [embase]/lim

**Web of Science (*n* ═ 427)**

TS ═ ((“budding” OR “sprouting” OR “bud” OR “buds” OR “tumor cell dissociation”) AND (“breast cancer”) AND (“mortality” OR “survival” OR “recurrence” OR “death” OR “prognosis” OR “progression” OR “metastasis”))

## Data Availability

All the data generated during the study was within the manuscript.
